# Integrating Network Pharmacology and Molecular Docking to Analyse the Potential Mechanism of action of *Macleaya cordata (Willd.) R. Br.* in the Treatment of Bovine Hoof Disease

**DOI:** 10.3390/vetsci9010011

**Published:** 2021-12-30

**Authors:** Zhen Dong, Mengting Liu, Xianglin Zou, Wenqing Sun, Xiubin Liu, Jianguo Zeng, Zihui Yang

**Affiliations:** 1 Hunan Key Laboratory of Traditional Chinese Veterinary Medicine, Hunan Agricultural University, Changsha 410128, China; 13104100291@163.com (Z.D.); liumengting@stu.hunau.edu.cn (M.L.); zouxianglin@stu.hunau.edu.cn (X.Z.); sun207444@163.com (W.S.); xiubin_liu@hunau.edu.cn (X.L.); 2 College of Veterinary Medicine, Hunan Agricultural University, Changsha 410128, China; 3 College of Animal Science and Technology, Hunan Agricultural University, Changsha 410128, China

**Keywords:** *M. cordata*, hoof disease, network pharmacology, molecular docking, mechanism of action

## Abstract

Based on network pharmacological analysis and molecular docking techniques, the main components of *M. cordata* for the treatment of bovine relevant active compounds in *M. cordata* were searched for through previous research bases and literature databases, and then screened to identify candidate compounds based on physicochemical properties, pharmacokinetic parameters, bioavailability, and drug-like criteria. Target genes associated with hoof disease were obtained from the GeneCards database. Compound−target, compound−target−pathway−disease visualization networks, and protein−protein interaction (PPI) networks were constructed by Cytoscape. Gene ontology (GO) analysis and Kyoto Encyclopedia of Genes and Genomes (KEGG) pathway enrichment analyses were performed in R language. Molecular docking analysis was done using AutoDockTools. The visual network analysis showed that four active compounds, sanguinarine, chelerythrine, allocryptopine and protopine, were associated with the 10 target genes/proteins (SRC, MAPK3, MTOR, ESR1, PIK3CA, BCL2L1, JAK2, GSK3B, MAPK1, and AR) obtained from the screen. The enrichment analysis indicated that the cAMP, PI3K-Akt, and ErbB signaling pathways may be key signaling pathways in network pharmacology. The molecular docking results showed that sanguinarine, chelerythrine, allocryptopine, and protopine bound well to MAPK3 and JAK2. A comprehensive bioinformatics-based network topology strategy and molecular docking study has elucidated the multi-component synergistic mechanism of action of *M. cordata* in the treatment of bovine hoof disease, offering the possibility of developing *M. cordata* as a new source of drugs for hoof disease treatment.

## 1. Introduction

Hoof disease is a common surgical disease in equine animals and cattle, with a higher incidence in dairy cattle [1]. In the past, cattle and horses, as the main livestock workforce, often suffered from hoof disease due to changes in the normal structure of the hoof, caused by heavy weight bearing and overwork [2,3]. Hoof disease is influenced by a number of factors, including genetics, physiological abnormalities, shed hygiene, feeding management, mechanical injuries, and infections by pathogenic bacteria, as well as feed ratios and metabolic diseases in cows [4]. Depending on the location of the hoof disease, it can be divided into diseases of the hoof cortex and dermis (hoof fissures, laminitis, and restricted hoof dermatitis), diseases of the interdigital space (interdigital dermatitis, rotten hoof disease, etc.), and diseases of the deep hoof tissue (arthritis, tenosynovitis, etc.). Hoof disease is often accompanied by increased hoof temperature, pain, and subsequent lameness, and cows can also suffer from secondary mastitis resulting in reduced milk production, which seriously affects animal welfare and the economic development of the livestock industry. In current production, hoof disease is mainly treated with non-steroidal anti-inflammatory drugs combined with antibiotics, but due to the high incidence of hoof disease and the long-term use of these drugs, they may produce a variety of side effects and resistance, and lead to a reduction in the effectiveness of treatment [5,6].

Chinese medicine, as the primary means of treating disease in Chinese veterinary medicine, has definite therapeutic effects and fewer side effects, and can be used as a complementary and alternative therapy to modern medicine. *Macleaya cordata (Willd.) R. Br.* (*M. cordata*), also called Boluohui, is a plant of the opium poppy family native to China, and is widely used in animal husbandry in Asia, Europe, and the Americas. Numerous studies have proven that *M. cordata* extract can eliminate intestinal inflammation, regulate intestinal flora, and promote growth in livestock [7,8,9,10]. In addition, *M. cordata* extract also showed good antibacterial effects and facilitated the elimination of bacterial resistance [11]. The Chinese Materia Medica records that *M. cordata* is used externally to relieve pain and swelling and to treat bruises and arthritis [12]. With the development of industrialized society, horses and cattle are no longer used as draft animals, and the number of horses kept has declined significantly. Conversely, intensive farming has led to a rapid expansion of beef and dairy cattle farming. Based on this, *M. cordata* has promising applications for the treatment of hoof disease in cattle. However, herbal treatments are multi-component, multi-pathway, and multi-target, and the mechanism of action of *M. cordata* in the treatment of arthritis has not been elucidated and needs further research.

Network pharmacology is based on an extension of pharmacology under systems biology and the application of technical tools such as databases and computational chemistry to study the targets, pathways, and efficacy of drugs. It can also be combined with molecular biology or a range of emerging histological tools, such as metabolomics and transcriptomics, to provide a macroscopic and systematic understanding of the interactions between drugs, diseases, and the organism from a holistic perspective [13]. In the past, Chinese (veterinary) medicine only emphasized the effectiveness and safety at a macro level, lacking mechanistic research on the pharmacology and toxicology, and the systemic characteristics were expressed in the philosophical approach [14]. The application of network pharmacology can better provide ideas for the modernization of Chinese (veterinary) medicine research and promote the transformation of Chinese (veterinary) medicine from traditional empirical medicine to modern evidence-based and precision medicine. Applying this model, there have been a number of successful attempts at research [15,16]. Therefore, this study was conducted to predict the targets of the active ingredients in *M. cordata* based on network pharmacology, to analyze the combined pharmacological effects of *M. cordata* at the network level for the treatment of bovine hoof disease, and to validate the interactions between the predicted compounds and the targets using molecular docking techniques, with a view to fully exploit *M. cordata* for wider use in veterinary practice.

## 2. Materials and Methods

Network pharmacology-based predictions for the treatment of hoof disease with *M. cordata* were constructed through the following steps: (1) data collection and collation, including identification of the chemical composition of *M. cordata*, screening of candidate compounds, identification of hoof disease-related disease targets, and intersection of the predicted targets of candidate compounds with the disease targets; (2) network topology analysis and protein interaction network construction; (3) enrichment analysis; and (4) molecular docking validation.

### 2.1. Data Collection

#### 2.1.1. Chemical Composition of *M. cordata*

According to a large amount of literature, it is known that the main active substances of *M. cordata* are alkaloids, based on literature search engines such as China National Knowledge Infrastructure (CNKI) (https://www.cnki.net/) (accessed on 26 October 2021), PubMed (https://pubmed.ncbi.nlm.nih.gov/) (accessed on 28 October 2021), and Chemistry Database (http://www.organchem.csdb.cn/) (accessed on 27 October 2021) [17] for related compounds.

#### 2.1.2. ADME Pharmacokinetic Evaluation

Traditional Chinese medicine is usually administered orally or externally, and a large number of chemicals enter the body and are involved in physiological processes such as absorption, distribution, metabolism, and excretion (ADME). The new drug development process, where approximately 90% of compounds are eliminated due to drug properties, has shown, in a large number of cases, that ADME properties are more critical in the screening process of new drugs than the pharmacological activity of the compounds themselves [18]. ADME testing of four candidate compounds was performed using the SwissADME tool (http://www.swissadme.ch/) (accessed on 30 October 2021) [19] and evaluated on the basis of three properties—water solubility, pharmacokinetic properties, and drug-like properties. The more water-soluble the substance, the weaker the ability to pass through the skin, depending on the conditions evaluated under each parameter. The pharmacokinetic properties in this screening focused on skin permeability (Log K*p*); the smaller the value, the less molecules are permeated by the skin. There are five indicators under the class of drug properties, two of which are yet to be included in the selection; with a bioavailability F > 10%, the higher the value, the better the bioavailability.

#### 2.1.3. Target Gene Prediction for the Selected Compounds

The Simplified molecular input line entry system (SMILES) of the selected compounds were queried using the PubChem database (https://pubchem.ncbi.nlm.nih.gov/) (accessed on 30 October 2021) [20], and then the SMILES of the compounds were entered into the SwissTargetPrediction web tool (http://www.swisstargetprediction.ch/) (accessed on 1 November 2021) [21]for target gene prediction. Gene symbol data were obtained from The Universal Protein Resource (UniProt) database (https://www.uniprot.org/) (accessed on 1 November 2021) [22].

#### 2.1.4. Target Gene Screening for Disease

Based on the presentation and site of hoof disease, the GeneCards^®^: The Human Gene Database version 5.5 database (https://www.genecards.org/) (accessed on 2 November 2021) [23] was searched for information on known therapeutic targets associated with arthritis, tenosynovitis, bursitis, laminitis, pain, and thrombus.

#### 2.1.5. Venn Analysis

For all of the target genes related with the chosen chemicals and hoof disease, a Venn diagram was constructed using R 4.0.3 (R Core Team, Vienna, Austria), demonstrating the intersection between compound predicted targets and disease known targets.

### 2.2. Network Topology Analysis and Protein Interactions Network Construction

#### 2.2.1. Network Topology Analysis

Visualization of compound−target networks and compound−target−pathway networks by Cytoscape 3.7.1 (https://github.com/cytoscape/cytoscape/releases/3.7.1/, accessed on 20 January 2019) and through systematic exploration of the active compounds and the possible molecular mechanisms for the treatment of hoof-like diseases by *M. cordata*.

#### 2.2.2. Protein Interaction Network Construction

The target genes obtained from the Venn analysis of intersections were imported into the Multiple Proteins text box in String database v11.5 [24] to construct a protein interaction network (PPI) in order to systematically understand the protein interactions in order to screen for the highest possible importance of proteins in the network. Before constructing the PPI network, the organism was set as a common bovine (*Bos taurus*), the minimum interaction score was set to “medium confidence (0.40), and the unconnected protein nodes were excluded. The constructed PPI network data were mapped using Cytoscape 3.7.1.

#### 2.2.3. Enrichment Analysis

Gene Ontology (GO) and Kyoto Encyclopedia of Genes and Genomes (KEGG) pathways were performed on the target genes using the DAVID Bioinformatics Resources 6.8 database (https://david.ncifcrf.gov/) (accessed on 4 November 2021) [25,26]. The species selected for the enrichment analysis was common bovine (*Bos taurus*). A false discovery rate (FDR) < 0.05 was considered statistically significant in the results of the enrichment analysis.

#### 2.2.4. Molecular Docking

The TOP5 proteins were screened for molecular docking validation based on the results of the PPI network. High resolution standard protein 3D structure pdb files were obtained from the RCSB Protein Data Bank database (https://www.rcsb.org/) (accessed on 5 November 2021) [27]. Compound conformation sdf files were obtained from PubChem, and the format was converted using the Open Babel tool to generate mol2 or pdb format files before the molecular docking analysis was performed using AutoDockTools 1.5.6, and the molecular docking results were generated after processing by PyMOL software [28]. The screening condition was set to select only the first three stable conformations with the lowest binding energy and binding energy ≤−5 kcal/mol, and the number of bound hydrogen bonds ≥2.

## 3. Results

### 3.1. Data Collection

#### 3.1.1. Screening of Compounds in the *M. cordata*

**Figure 1:**
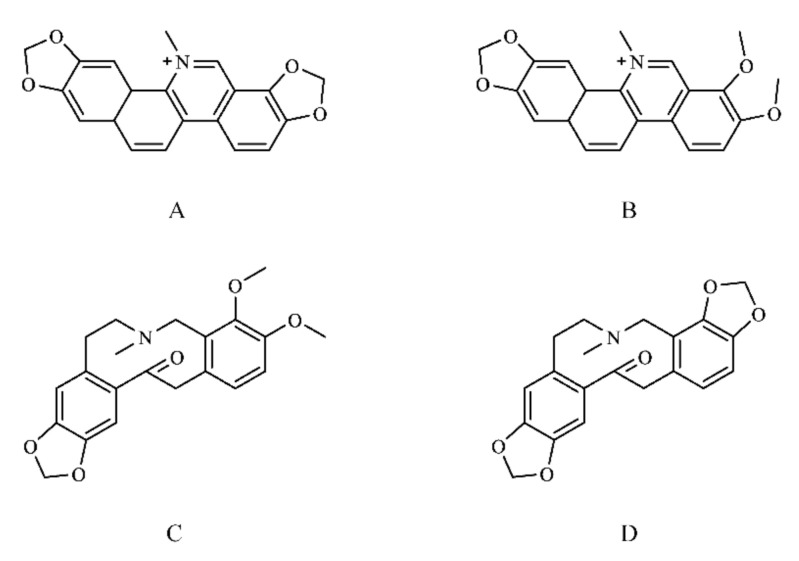
Chemical structures of the candidate compounds. (**A**) Sanguinarine; (**B**) Chelerythrine; (**C**) Allocryptopine; (**D**) Protopine.

Four candidate compounds were screened based on the alkaloid content in *M. cordata* and an extensive pre-laboratory base [29,30], namely Sanguinarine (SAN), Chelerythrine (CHE), Allocryptopine (ALL), and Protopine (PRO) (Figure 1).

#### 3.1.2. ADME Screening and Associated Target Gene Prediction

The information related to the four compounds evaluated by ADME is shown in Table 1. All four candidate compounds had a molecular weight less than 600, water−oil partition coefficient (Log P_o/w_) of 0–3, with moderate water solubility and skin permeability of −5 to −7 cm/s. All of the drug-like parameters were met and the bioavailability was 0.55. All four candidate compounds were eligible by evaluation screening; 161 compound-related genes were obtained in the UniProt database through the SwissTargetPrediction tool.

#### 3.1.3. Disease Target Gene Acquisition

A total of 24,465 target genes associated with six diseases, including arthritis and tenosynovitis, were retrieved from the GeneCards database, including 12,456 for pain, 358 for tenosynovitis, 77 for bursitis, 7146 for arthritis, 3487 for laminitis, and 941 for thrombus. After eliminating 20,411 genes by correlation scoring and repeatability screening, 4054 disease target genes were retained.

#### 3.1.4. Genetic Intersection of Compounds and Disease Targets

**Figure 2:**
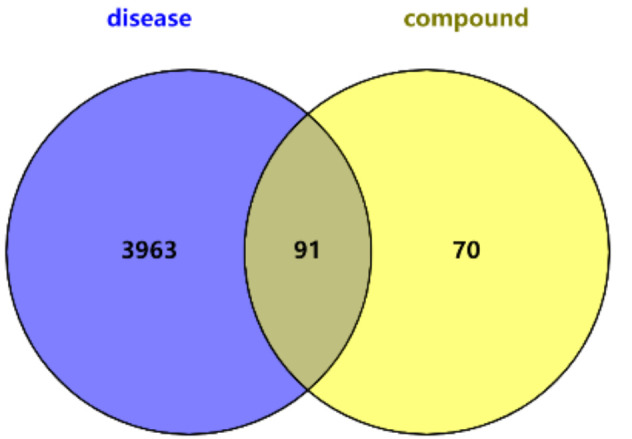
Venn diagram identifying disease and compound target intersections.

A total of 91 genes were obtained by intersecting 161 compound predicted target genes with 4054 disease target genes by R 4.0.6 (Figure 2).

### 3.2. Network Topology Analysis and PPI Network Construction

#### 3.2.1. Network Topology Analysis

**Figure 3:**
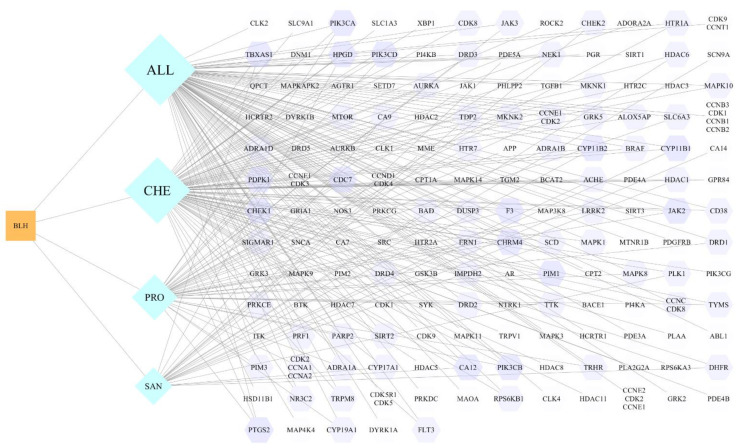
Compound−target interaction network diagram (orange square represents plant, blue diamond represents compound, and purple octagon represents target point, BLH: Boluohui; SAN: Sanguinarine, CHE: Chelerythrine, All: Allocryptopine, PRO: Protopine).

The constructed network of compounds and their potential target interactions is shown in Figure 3. The network consists of 166 nodes (1 plant, 4 compounds, and 161 targets) and 253 edges, where the orange nodes represent the plant *M. cordata*, the light blue nodes represent the compounds, and the lavender nodes represent the interactions. Table 2 shows the degree and betweenness of the four main active compounds in the *M. cordata*. The higher the degree value of a node, the higher its degree centrality, meaning the node is more important in the network, and the betweenness describes the node importance in terms of the number of shortest paths through the node [31]. ALL has the most targets of action with 101, followed by CHE with 89, an PRO and SAN have 41 and 23 targets, respectively. The network properties suggest that ALL may be the most dominant substance at play.

**Figure 4:**
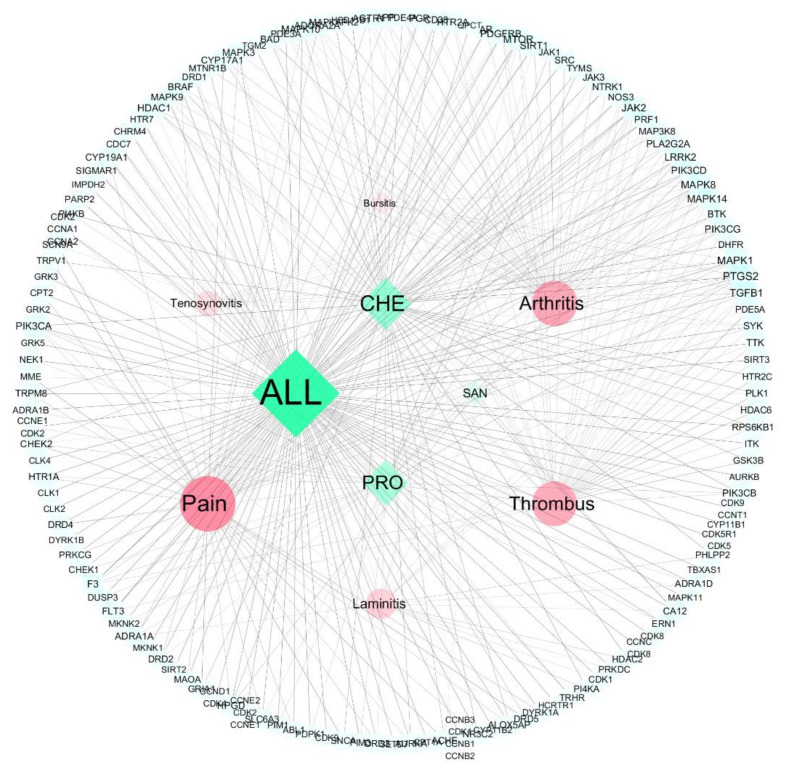
Compound−target−hoe disease network diagram (green diamonds for compounds, red circles for diseases, blue squares for targets).

**Figure 5:**
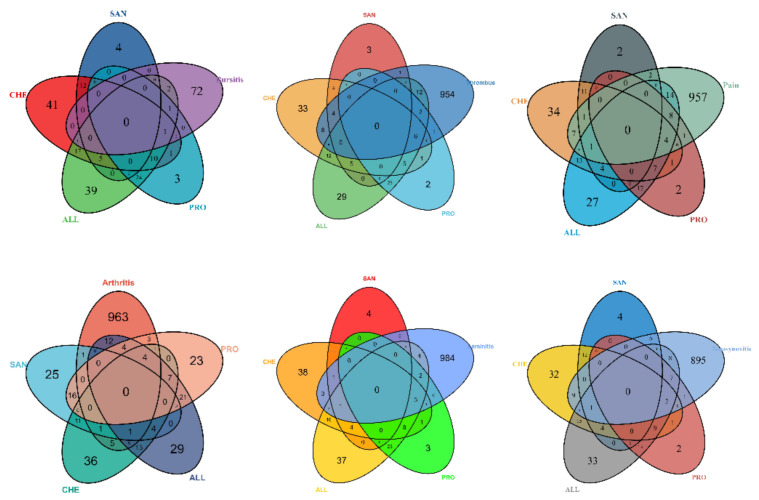
Venn diagram of target genes between compounds and hoof disease-related diseases.

The compound−target interaction network was mapped to the disease network to construct a compound−target−disease interaction network graph (Figure 4). The network consisted of 145 nodes and 371 edges, and Figure 5 demonstrate the target intersections of the four active compounds in the *M. cordata,* with the six diseases associated with hoof disease. Of these, ALL had 32, 26, and 26 targets associated with pain, arthritis, and thrombosis, respectively; CHE had 18 targets associated with pain, 18 with thrombus, and 16 with arthritis; the number of intersecting targets for pain, arthritis, and thrombus with PRO was 13, 12, and 9, respectively; and SAN had fewer target genes associated with all six diseases, with the most being associated with thrombus at 5.

#### 3.2.2. PPI Network

**Figure 6:**
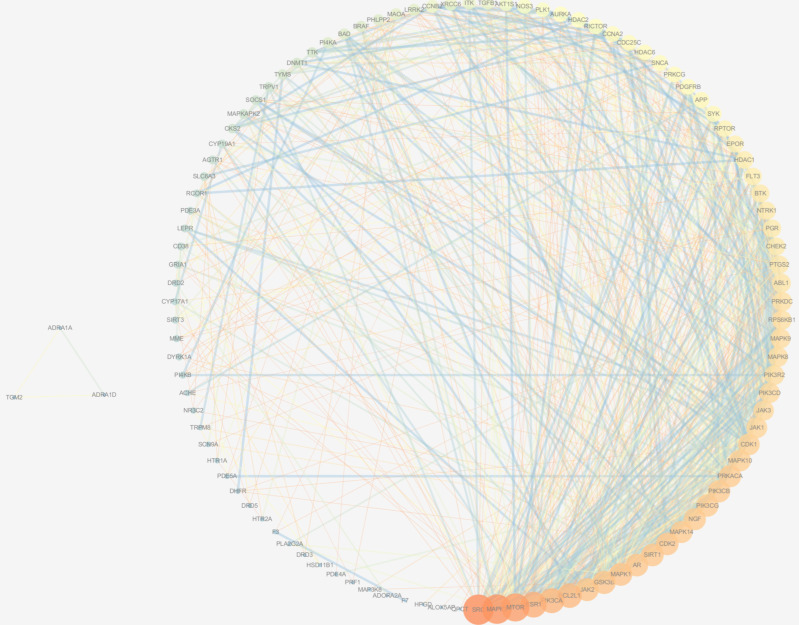
Protein−protein interaction network.

The nodes in the PPI network represent proteins and the edges represent the interactions between proteins. The constructed network (Figure 6) has 106 nodes and 831 edges. The nodes in the network were ranked according to the magnitude of the degree value and the top 10 proteins with the highest connectivity were selected, including cell growth, proliferation, and differentiation-related SRC, MAPK3, MTOR, MAPK1, GSK3B, and JAK2; hormone-related ESR1 and AR; and apoptosis-related PIK3CA, BCL2L1, and SIRT1. The protein network attributes are described in Table 3.

### 3.3. Enrichment Analysis

#### 3.3.1. GO Enrichment Analysis

**Figure 7:**
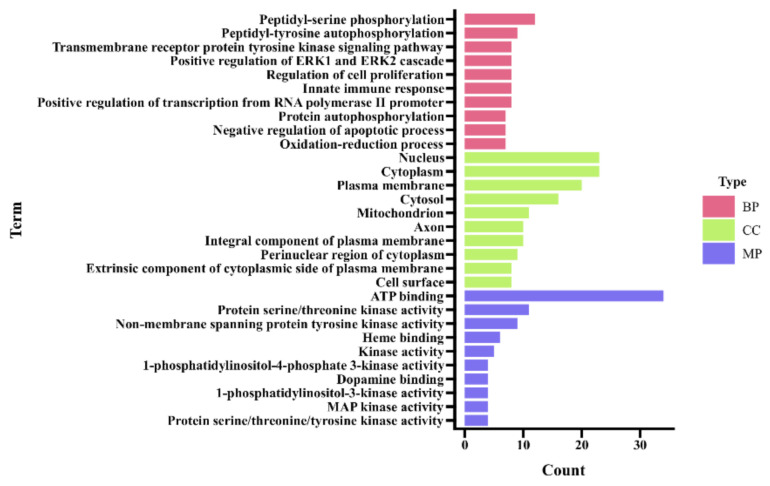
Gene Ontology (GO) enrichment analysis of the targets. (BP: biological processes, CC: cellular components, MF: molecular functions).

The GO enrichment analysis included three biological aspects, namely biological processes (BP), cellular components (CC), and molecular functions (MF). As shown in Figure 7, the bar chart shows the top 10 enrichment terms for each biological aspect. The results show that many targets are involved in a variety of BPs associated with immune responses and inflammatory responses, such as protein phosphorylation, innate immune response, and regulation of apoptotic signaling pathways, confirming the relevance to the pathogenesis of hoof disease. The CC results show that most targets are localized in the cell membrane and cytoplasmic fractions. The MF results show that most targets are associated with enzyme activity.

#### 3.3.2. KEGG Pathway Enrichment

**Figure 8:**
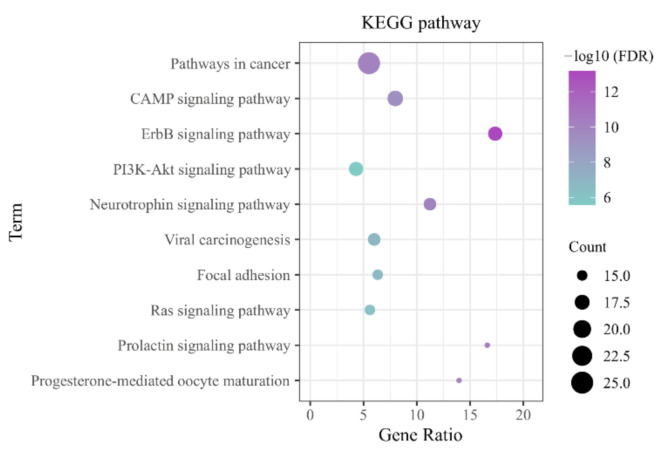
KEGG pathway enrichment analysis of the targets.

**Figure 9:**
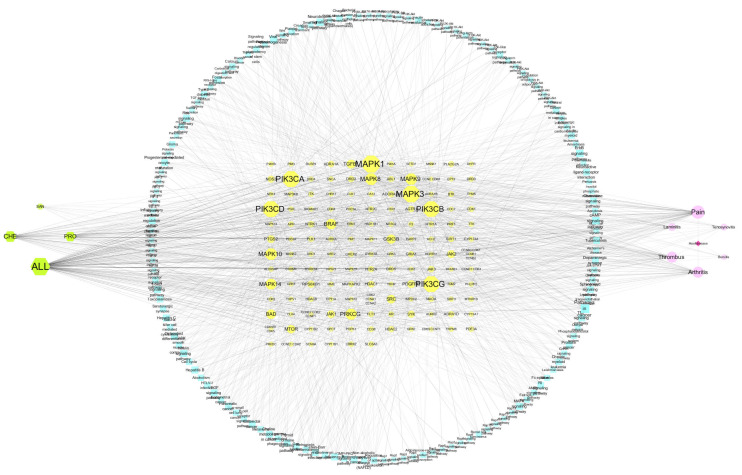
Compound−target−pathway−disease interaction visualization network.

KEGG pathway analysis was used to identify the target functions and signaling pathways, and the bubble diagram (Figure 8) demonstrates the top 10 potential pathways (FDR < 0.05), while a compound−target−pathway−disease visualization network was constructed (Figure 9). The results show that most of the pathways are involved in cell proliferation and differentiation, hormone secretion, and angiogenesis, and are closely associated with the pathogenesis and prognosis of diseases such as arthritis.

### 3.4. Molecular Docking

**Figure 10:**
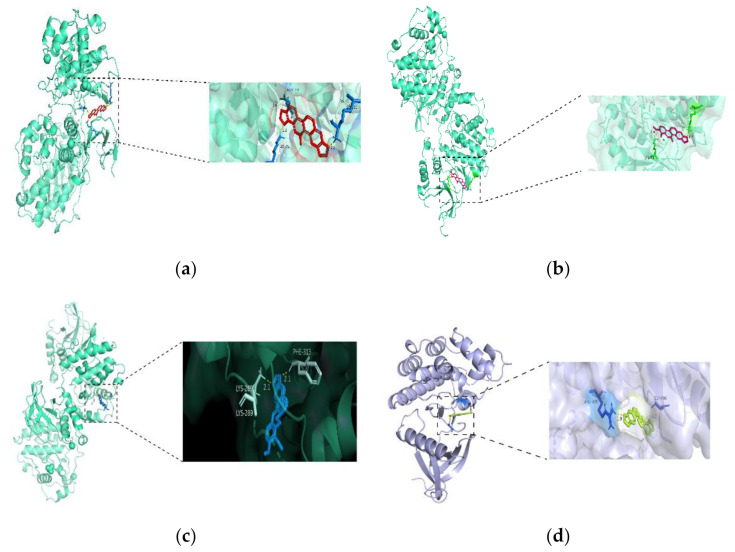
Molecular docking results: (**a**) SAN and MAPK3; (**b**) CHE and MAPK3; (**c**) ALL and MAPK3; (**d**) PRO and JAK2.

The four active compounds were semi-flexibly docked with the top 10 protein targets in the PPI interaction network using AutoDockTools, with the Nice Level set to 20, and four results were obtained that met the screening criteria, namely SAN-MAPK3, CHE-MAPK3, ALL-MAPK3 and PRO-JAK2. The docking parameters are shown in Table 4, and the mode of action is shown in Figure 10.

## 4. Discussion

*M. cordata* extract has good anti-inflammatory and growth-promoting effects and is now widely used in livestock production. In traditional Chinese medicine, *M. cordata* is used as an external medicine for bruises and arthritis, but the mechanism of action has not been elucidated. In relation to veterinary clinical and animal production, hoof disease is a common surgical disease in cattle, horses, and other animals. Based on the efficacy of empirical medicine and the rational use of medicinal plant resources, *M. cordata* has potential therapeutic effects and is expected to be developed as a new source of drugs for the treatment of hoof disease.

Traditional drug development follows a reductionist approach based on molecular biology, but it is difficult to reveal the systemic characteristics of traditional Chinese medicine (TCM). Network pharmacology can resolve the complex system of multi-component, multi-target, and multi-pathway of TCM at the systemic level, which is of guidance for both active ingredient screening and target discovery of TCM [32]. For example, Liao et al., based on the principle of drug combination of compound Dan Shen tablets, screened the main active substances, tanshinol and borneol, through network pharmacology and synthesized a novel compound, tanshinol borneol ester (DBZ), for the treatment of cardiovascular and cerebrovascular diseases [33].

In this study, four alkaloid active compounds, SAN, CHE, ALL, and PRO, were screened based on previous studies, which have shown the compounds to have good antibacterial and anti-inflammatory pharmacological effects [34,35,36]. When screening for ADME, it is generally accepted that compounds with a molecular weight greater than 600 are difficult to be absorbed through the skin; and the more water-soluble the substance, the more difficult it is to pass through the skin (Log P < 0); strongly lipophilic substances (Log P > 3) can easily pass through the skin barrier but are difficult to be absorbed when they enter the body, and small molecules can penetrate through the skin and are well absorbed when the water−oil partition coefficient is between 0 and 3. In addition, skin permeability (Log K*p*) also determines whether a drug can pass through the skin into the body; at Log K*p* < −10, the substance is essentially unable to pass through the skin [37,38]. According to some previous pharmacokinetic studies, the half-lives of the four main components are short, with the most persistent compound being SAN (unpublished) [39,40]. In summary, all four compounds exhibited physicochemical properties suitable for transdermal administration, demonstrating the efficacy of the in vitro administration of *M. cordata*. Toxicity and/or adverse effects are always a concern during the development of a drug. Although the results of the cytotoxicity tests showed a high cytotoxicity of SAN and CHE [41,42]. However, the lack of results in animal studies did not show corresponding results [42,43]. In addition, due to the special barrier function of the skin, transdermal drug delivery resulted in a more stable drug concentration and low toxicity to the affected area. In clinical applications, more toxicity issues depend on the dosage form [44]. *Chelidonium majus L*, which has a similar active ingredient to *M. cordata*, has been used by EMA in the treatment of skin conditions such as tissue healing [45,46]. However, skin damage has been reported with excessive abuse of pharmaceutical products containing high levels of SAN [47]. Therefore, more emphasis should be placed on safe concentrations and formulation studies in drug development and use.

A total of 161 potential targets associated with compounds and 4054 gene targets associated with hoof disease were identified, and 91 target genes were obtained from targets where the compounds interacted with hoof disease. The proteins that play a key role were obtained through the PPI network, and the GO and KEGG pathway enrichment analyses showed evidence of bioregulatory processes such as cell proliferation and differentiation, apoptosis, and hormone secretion associated with hoof disease. The good binding ability of ALL-MAPK3 and PRO-JAK2 was illustrated by rigorous molecular docking.

The top 10 proteins in the PPI network in terms of connectivity all play an important role in the course of hoof disease. Of these, SRC was the most highly associated and was shown to bind to PTK2B/PYK2 to provide energy for anti-osteoporosis drugs and to prevent the inhibitory effect of calcitonin by activating mitochondrial cytochrome C oxidase [48]. SRC can also regulate IL-6 by activating the YAP1-NOTCH pathway and is abundantly expressed in response to epithelial mucosal injury, promoting epithelial healing and maintaining barrier function [49]. SRC family tyrosine kinases inhibit platelet recruitment, providing additional antithrombotic efficacy and improving circulation [50]. MAPK1/ERK2 and MAPK3/ERK1 are two MAPKs that play an important role in the MAPK/ERK cascade response and can regulate a variety of biological functions such as cell growth, adhesion, and differentiation through the regulation of transcription, translation, and cytoskeletal rearrangement, and numerous studies have demonstrated that MAPK plays a key role in skeletal development and inflammation. Mechanical stimuli such as traumatic friction and fractures can induce an increased expression of Fak through activation of the Erk, Jnk, and p38-MAPK pathways, and activate the Fak-MAPK pathway to promote bone formation and regeneration [51], and p38-MAPK is also a key signaling molecule in the treatment of inflammatory diseases [52]. Yu et al. found that bone loss caused by certain inflammatory diseases can stimulate LL-37 expression and suppress inflammation and promote osteoclastogenesis via the P2X7R and MAPK signaling pathways [53]. Dai et al. found that curcumin alleviated inflammation and synaptic proliferation induced by arthritis in rats via the mTOR pathway [54]. In conclusion, elevated levels of cellular inflammatory factors are thought to drive a critical component of the bone inflammatory disease process, and the high expression of inflammatory factors typically activates a number of signaling pathways, including Janus kinase/signal transducer and activator of transcription protein (JAK/STAT), stress-activated protein kinase/mitogen-activated protein kinase (SAPK/MAPK), and phosphatidylinositol-3-kinase/protein kinase B/rapamycin mechanistic target (PI3K/Akt/mTOR) pathways that regulate a large number of cellular responses [55,56,57], and most of the top 10 proteins screened function in these signaling pathways. In addition, BCL2L1 can reduce the effect of inflammation on NLRP1 inflammatory vesicle activation, thus affecting CASP1 activation and IL1B release, involved in inflammatory and apoptotic processes [58]. Hormone receptors (AR and ESR1) are even more important players in the inflammatory process and can play a potent role in bone inflammatory diseases such as arthritis and bursitis, through the synthesis of steroid hormones (sex hormones and glucocorticoids) via the second messenger cAMP pathway [59,60].

Key proteins in the cAMP, PI3K-Akt, Prolactin, and Ras pathways and PPI networks obtained from KEGG pathway enrichment analysis interact with each other to form a complex target−pathway network. For example, the downstream proteins of the Prolactin pathway are JAK2, MAPK, and SRC and regulate apoptosis and inflammation through the JAK/STAT pathway [61,62,63]. MAPK integrates crosstalk with multiple signaling pathways, such as the Ras/MAPK pathway, ErbB pathway, and PI3K-Akt pathway. Together, these signaling pathways play a role in osteoblast growth, proliferation, apoptosis angiogenesis, and inflammation regulation [64,65,66,67,68,69]. There has been some evidence of a therapeutic role for SAN in hoof disease-related disorders. SAN can act on PI3K-Akt-GSK3β and Src (Tyr-416) to inhibit platelet activation and reduce thrombosis [70]. SAN inhibits VEGF-induced Akt phosphorylation to exert anti-angiogenic effects and plays an important role in arthritis [71,72]. Furthermore, SAN has also been shown to have pain-relieving (through inhibition of the MAPK signaling pathway) and wound-healing properties that could help treat hoof disease and improve animal welfare. [73,74,75]. SAN’s potential role in protecting osteoblasts, preventing osteoarthritis, and regulating osteophytes is also closely linked to the prevention and prognosis of hoof disease [76,77,78].

The semi-flexible molecular docking results showed good binding of SAN to MAPK3, CHE to MAPK3, ALL to MAPK3, and PRO to JAK2. The results of molecular docking are consistent with the reported results. These targets with a high affinity to the compounds are likely to be the key to the treatment of hoof disease with *M. cordata*, in particular, the MAPK pathway may play a very relevant role.

## 5. Conclusions

In summary, *M. cordata* has the potential to treat hoof diseases, where the therapeutic effects of the four main active substances, SAN, CHE, ALL, and PRO, are related to targets such as SRC, MAPK3, JAK2, and MTOR, and act through signaling pathways such as cAMP, JAK/STAT, PI3K-Akt, and Ras/MAPK. This shows that the treatment of hoof disease-related diseases by *M. cordata* is the result of the interaction of multiple components, multiple targets, and multiple pathways.

## Figures and Tables

**Table 1:** The results of the absorption, distribution, metabolism, and excretion (ADME) evaluation of candidate compounds.

Compounds	Molecular Weight	Water-Oil Distribution Factor	Estimated SOLubility (ESOL) Class	Skin Penetration	Drug-Like Properties (Number of Yes/5)	Bioavailability
Sanguinarine (SAN)	332.33	2.88	Moderately soluble	−5.17	5/5	0.55
Chelerythrine (CHE)	348.37	3.02	Moderately soluble	−5.17	5/5	0.55
Allocryptopine (ALL)	369.41	2.81	Moderately soluble	−6.48	5/5	0.55
Protopine (PRO)	353.37	2.67	Moderately soluble	−6.47	5/5	0.55

**Table 2:** Network parameters of the compounds.

Compounds	Degree	Betweenness
SAN	22	16,525.8
CHE	89	15,077.4
ALL	101	2489.3
PRO	41	1679.5

**Table 3:** Network properties of the top 10 proteins with protein-protein interaction (PPI) network degree values.

UniProt Entry	Gene Symbol	Protein Name	Degree	Betweenness
P12931	SRC	SRC proto-oncogene, non-receptor tyrosine kinase	49	712.8
P27361	MAPK3	mitogen-activated protein kinase 3	47	620.4
P42345	MTOR	mechanistic target of rapamycin kinase	44	478.5
P03372	ESR1	Estrogen receptor	39	459.0
P42336	PIK3CA	Phosphatidylinositol 4,5-bisphosphate 3-kinase catalytic subunit alpha isoform	37	203.3
Q07817	BCL2L1	Bcl-2-like protein 1	36	266.0
P28482	MAPK1	Mitogen-activated protein kinase 1	33	169.5
P49841	GSK3B	Glycogen synthase kinase-3 beta	33	438.6
O60674	JAK2	Tyrosine-protein kinase JAK2	33	154.9
P10275	AR	Androgen receptor	32	271.3
Q96EB6	SIRT1	NAD-dependent protein deacetylase sirtuin-1	32	419.3

**Table 4:** Molecular docking parameters.

	Combination of Energy (kcal/mol)	Number of Hydrogen Bonds
SAN-MAPK3	−6.98	3
CHE-MAPK3	−7.50	3
ALL-MAPK3	−8.62	2
PRO-JAK2	−6.58	2
